# Enhancing Volleyball Athlete Performance: A Comprehensive Review of Training Interventions and Their Impact on Agility, Explosive Power, and Strength

**DOI:** 10.7759/cureus.53273

**Published:** 2024-01-31

**Authors:** Ashish A Keoliya, Swapnil U Ramteke, Manali A Boob, Kamya J Somaiya

**Affiliations:** 1 Sports Physiotherapy, Ravi Nair Physiotherapy College, Datta Meghe Institute of Higher Education and Research, Wardha, IND

**Keywords:** sports performance, sports rehabilitation, volleyball, speed and agility, agility

## Abstract

Volleyball is one of the most globally renowned sports in terms of global popularity. The game is a team sport that both men and women can participate in. The gameplay relies heavily on physical activities such as jumping, landing, and quick movements, often causing strain on the musculoskeletal system and leading to injuries. For this reason, agility training is crucial to improving a player's ability to change direction swiftly as and when required by the gameplay. Although it is relatively safer than other team sports, actions like jumping, blocking, and spiking can lead to potential injuries. Properly monitoring the training loads and injury prevention during training should be the major focus in formulating a holistic training methodology in volleyball training. The main goal of this literature study is to evaluate the impact of various training interventions on agility and other performance parameters specific to volleyball players. The range of research approaches and interventions described in this literature review highlights the significance of agility in volleyball training. In many studies, the use of tailored training programs for volleyball has been shown to have positive effects on agility, strength, and jump performance. Although there are limitations to the study design and sample size, the findings from this review necessitate the need for better scientifically informed training programs to reduce injury risk while enhancing player's overall performance potential. To conclude, the current literature review highlights the importance of agility training in volleyball, providing insights into effective training strategies and highlighting the low quality of evidence, suggesting the need for well-structured research on the topic.

## Introduction and background

Volleyball is one of the most globally popular sports, played by approximately 800 million people worldwide [1-3]. Sports performance, particularly in sports games, combat sports, and volleyball, relies primarily on fundamental components such as complex reaction speed, acceleration, maximum speed, whole-body change of direction, and agility [4-5]. Volleyball-specific activities, such as running, jumping, and landing on the ground, the ball blocks, and spikes, must be combined with agility and fast movements, putting a strain on the musculoskeletal system [6]. As a result, volleyball players are vulnerable to musculoskeletal problems [7]. Agility training is focused on improving an individual's ability to change their movement direction quickly [8,9]. It involves lateral movements, forward and backward jumps, and crisscross exercises [10]. Agility training in volleyball focuses on improving a player's ability to quickly change direction and respond to directional cues, enhancing their on-court performance by optimizing coordination between the central nervous system and proprioceptive feedback [11,12].

Although volleyball is generally seen as a safe activity, the overall risk of factors predisposing to injury mostly refers to body height, age, league, court position, proper warmup, and previous injury as reported by earlier findings [13]. Injuries in the sport of volleyball often result from actions such as jumping and landing, as well as from interactions with the ball during hitting and blocking [14]. The ball's high speeds, which can reach up to 80 mph, pose a significant risk of injury if it contacts a player's unintended body area. Specific positions on the volleyball court are linked to particular injury risks, with hitting and blocking being more injury-prone compared to passing or setting [15,16]. The majority of injuries, whether they are sudden and acute or develop over time due to overuse, occur during the physical act of jumping [17]. Overuse injuries tend to be more prevalent than acute injuries and are typically attributed to factors like improper technique, the volume of repetitive movements, or the playing surface's characteristics [18]. Elite athletes who dedicate more time to practice and training face a heightened risk of overuse injuries. This is likely due to their increased practice hours and intensity [19].

In the realm of current technological advancements, every sport undergoes rigorous scientific testing and training methodologies aimed at enhancing athletic prowess. Any metric employed becomes a valuable tool for assessing physical exercise and delving into the scientific aspects of sports performance [20,21]. Many athletes, trainers, and support personnel are adopting a more scientific approach to planning and monitoring training programs [22]. Monitoring training loads effectively is critical to measuring an athlete's adaptation to their training regimen and limiting potential risks associated with ineffective overloading, illness, or injury [23]. Various indicators are available to get insight into the influence of training load on athletes [24]. The training regimen in volleyball should be designed to align with the customary requirements of match intensity, the distinctive attributes of individual player specializations within the team's game strategy, and the unique characteristics of each athlete, and concurrently, it should be in line with the evolving trends of contemporary volleyball and the training methodologies [25-27]. Nonetheless, only a few of these indications are supported by solid scientific evidence, and a single, unambiguous marker in the existing corpus of literature on volleyball has yet to emerge [24].

In conclusion, while volleyball is a globally popular sport, it is critical to recognize and treat the inherent dangers, particularly those connected to injuries. A systematic and scientifically informed training program is critical for athletes to reduce these hazards and improve their performance. To ensure the long-term success and well-being of volleyball fans, players, coaches, and support staff must take a methodical approach based on evolving insights into the sport's unique demands.

## Review

Literature search strategy

**Figure 1:**
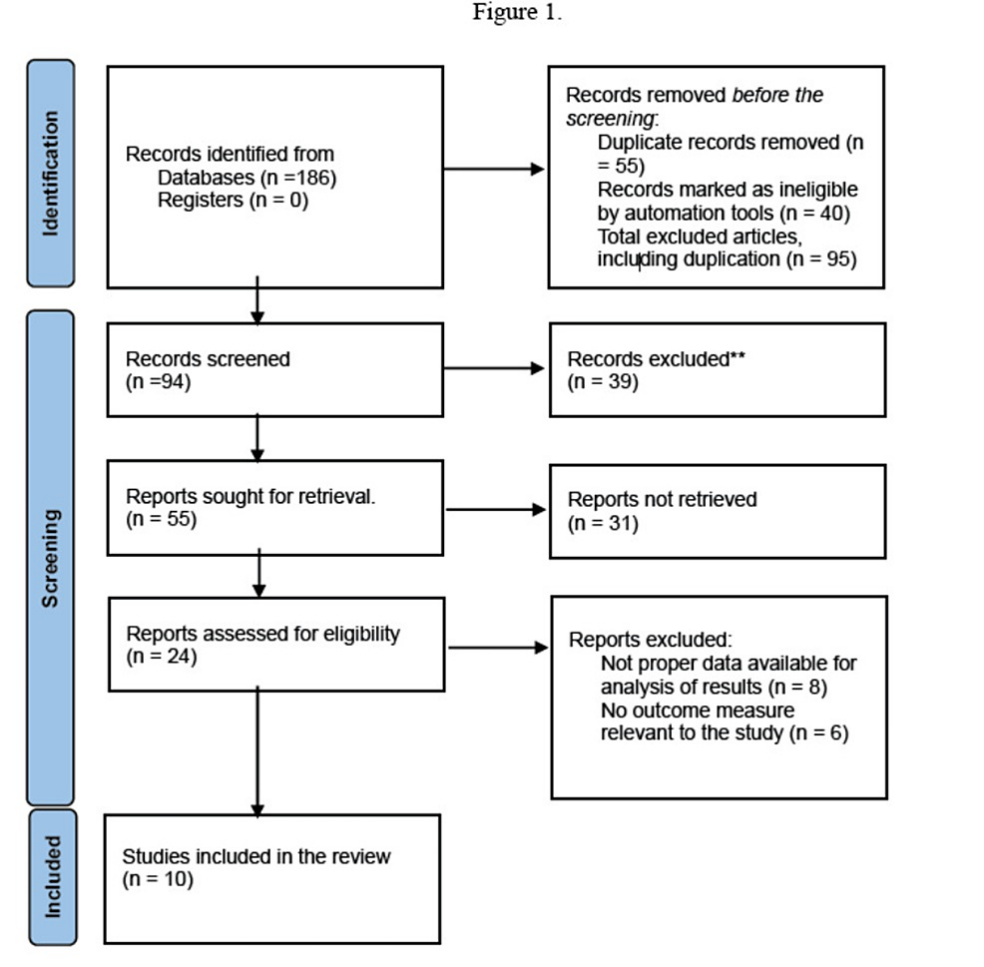
PRISMA chart PRISMA: Preferred Reporting Items for Systematic Reviews and Meta-Analyses

A precise search of works of literature in English-language literature from electronic databases was performed methodologically in accordance with a protocol for this review. All co-authors mutually agreed upon it. Four different databases, including PubMed, Physiotherapy Evidence Database, Semantic Scholar, and Google Scholar, were searched from 2012 to 2023. A total of 186 literature searches for papers were carried out using an appropriate combination of the keywords agility, agility training, and volleyball players. The reference lists of the included studies and pertinent review papers were screened as a secondary search. Out of these, 10 articles were available for review, as shown in Figure 1.

Study selection

The initial search yielded 186 studies (including 34 identified through a reference list search). EndNote software (Clarivate Analytics, Philadelphia, United States) was used to import the list of main articles, and duplicates were removed. Based on study design, intervention, participant characteristics, and sample size, 10 articles were selected for review, and titles and abstracts of the relevant articles were analyzed manually.

Types of outcome

A diverse set of outcome measures was utilized across the studies, evaluating aspects of athletic performance like agility, strength, power, and jump performance. The interventions implemented in the studies exhibited a wide spectrum, encompassing traditional resistance training, plyometric exercises, innovative methods such as dance video games, and structured programs like the International Federation of Association Football (FIFA 11+). These varied outcome measures and interventions underscore the multifaceted character of athletic performance in the context of volleyball. The findings emphasize the importance of tailored training approaches to address the specific needs and aspects of performance within the sport.

Data synthesis and analysis

For manual screening of the studies, the following inclusion criteria used were full-text publication from an English-language peer-reviewed journal. Participants were volleyball athletes. The experimental trial included pre- and post-testing, and at least one of the control groups received no intervention or an alternate exercise-based training program, an exercise training intervention that incorporated parts of training with a component relating to agility (balance or direction and velocity change), a six-week exercise training regimen with at least two weekly training sessions. The exclusion criteria were used for hand-screening of research, including non-athletic patients and articles in languages other than English, duplicate articles and non-full-text papers, review papers, systematic reviews, editorials, letters to the editor, comments, conference summary abstracts, and case reports.

Literature review matrix

Table 1 shows details of the articles included.

**Table 1:** Summary of the articles that are reviewed for agility training DAT: Decision-action time; CMJ: Countermovement jump; STG: Shuttle-run training group; ATG: Agility t-test performance; TRT: Traditional training; CRT: Cluster resistance training; RM: Repetition maximum; AVJ: Approach vertical jump; ITTA: Intention-to-treat analysis; BVJ: Block vertical jump

Authors and year of publication	Study type/methodology	Study sample	Intervention	Results	Conclusion
Chuang et al. 2022 [28]	Randomized controlled trial	27 young female volleyball players	Shuttle-run training group and agility-ladder training	Significant improvements were observed in decision-action time (DAT), agility T-test performance, and ten-meter sprint times (10 MS) for both the STG and ATG groups, indicating positive and statistically significant impact on the athletic performance of the treatment groups (STG and ATG) but not the control group.	The research yielded notable enhancements in vertical jump heights and agility times for young female volleyball athletes following an eight-week regimen that integrated both strength training and plyometric/agility conditioning.
Cakir and Ergi̇n 2022 [29]	Randomized controlled trial	20 young female volleyball players	Eight weeks of core training	For countermovement jump (CMJ), scores had a significant impact (F(1,18) = 24.01, p < 0.001). Vertical jump (VJ) scores, the analysis revealed a significant common effect of group and measurement time factors (F(1,18) = 7.038, p < 0.016). The same pattern was observed for the pro-agility test.	In conclusion, including core training in regular volleyball training improves balance, agility, and explosive strength. interconnected training approaches are critical for improving various performance qualities, highlighting the significance of augmenting regular volleyball training.
Ci̇N et al. 2021 [30]	Randomized controlled trial	28	Traditional training vs cluster resistance training	In contrast to traditional training, the cluster training group demonstrated notably greater improvements in the following domains: one-repetition maximum (1RM) back squat 1RM power output 1RM deadlift 1RM bench press	Cluster resistance training (CRT) offers superior benefits compared to traditional resistance training (TRT) for professional volleyball players. Utilizing CRT instead of TRT may enhance maximal strength, short sprint performance, agility, and vertical jump during the general preparation phase for professional volleyball players.
Hosseini 2019 [31]	Quasi-experimental study	24 male volleyball players	The FIFA 11+ training group and the control group	Eight weeks of 11+ Training significantly improved agility.	11+ training can help male college athletes enhance their agility and explosive power.
Hale 2019 [32]	Pre-post intervention design	15 female youth volleyball athletes	Eight-week combined strength and plyometric/agility offseason conditioning program. The first four weeks of the program focused on strength-building exercises, while the second four weeks were dedicated to power-based plyometric and agility exercises.	The height of the three vertical jump protocols exhibited significant increases with medium effect sizes after the eight weeks of offseason conditioning.	The researchers' hypothesis was validated as the vertical jump heights and agility times of young female volleyball athletes showed significant improvement following an 8-week off-season conditioning program that combined strength and plyometric/agility training.
Roopchand-Martin et al. 2018 [33]	Randomized pilot study	27 elite volleyball players	Video game dance training with ladder drills	The video game dancing group showed significant improvement in agility scores in both intention-to-treat and per-protocol analyses. In contrast, the ladder drills group showed no significant change in agility using intention-to-treat analysis but improved in per-protocol analysis.	This pilot study found that elite volleyball players' agility scores improved with video game dance training, indicating that more research is needed to investigate the significance of dance video gaming as an agility training technique.
Ho et al. 2016 [34]	Randomized controlled trial	26 volleyball players	Plyometric training	The results indicated improvements in the countermovement jump test and blocking agility test in the plyometric group	Plyometric training can increase the pace of force development for vertical leaps. Furthermore, it can significantly increase volleyball players' general agility, especially their lateral movement speed and quickness. This increase in mobility enables players to execute blocking moves quickly and efficiently.
Jastrzbeski et al. 2014 [35]	Randomized controlled trial	20	Plyometric high and low-intensity training	While both high-intensity and low-intensity jump training led to improvements in vertical jump heights and power output in certain tests, the specific context of explosive power during jump attacks or blocks did not exhibit statistically significant changes within the given experimental period for either group.	The study recommends that coaches determine exercise volume and intensity with a focus on individualization. In this particular study, training loads were personalized based on the players' mass and height. The study also highlights the importance of considering the total work rate, which varied among the groups and affected program effectiveness. Such an approach to Training is uncommon, and experiments of this nature help coaches better comprehend the impact of training methods on player performance. The training programs described in the study are suitable for both amateur and professional volleyball players
Gortsila 2013 [36]	Randomized controlled trial	45	10-week volleyball training program on different training surfaces (hard and sand)	Both the agility T-test and 505-test demonstrated significant improvements (p < 0.001) in all three groups, with Group S exhibiting significantly greater agility improvement compared to Groups I and C, regardless of the training surface	Training on sand surfaces could be a useful and effective tool for improving agility and passing skills in prepubescent female volleyball players
Stojanović and Jovanović 2012 [37]	Experimental research design	38 cadet volleyball players	Individual plyometric training programs were constructed	Following a six-week trial program, participants in the experimental group showed statistically significant increases in jumping agility. The findings suggest that the plyometric training program had a substantial impact on the development of explosive leg strength, which improved jumping agility.	A six-week plyometric training program has been shown to have a statistically significant effect on increasing explosive leg strength and, as a result, jumping agility. We advocate individually tailored plyometric programs as a more effective method for developing jumping agility in cadets.

Discussion

The systematic review examines multiple research that investigates the impact of various training programs on volleyball players' athletic performance. These studies, which use randomized controlled trials, quasi-experimental methods, and prospective experimental designs, examine characteristics such as sprint performance, agility, strength, and vertical leap. The aggregate findings provide useful insights into the efficacy of various training programs for improving volleyball players' athletic ability.

Chuang et al. (2022) conducted a randomized controlled experiment and found significant improvements in decision-action time, agility T-test performance, and 10-meter sprint times for shuttle-run and agility ladder training groups. This shows that these training strategies improve athletic performance in young female volleyball players [28]. Similarly, Cakir and Ergi̇n (2022) demonstrated, through a randomized controlled trial on core training, a significant impact on countermovement jump, vertical jump, and pro-agility test performance, highlighting the value of core training in developing explosive power and agility in female volleyball players [29]. Ci̇N et al. (2021) found that cluster resistance training (CRT) outperformed conventional resistance training in terms of strength gains across many measures. This highlights the potential benefits of CRT for total strength development, which is an important component of volleyball performance [30].

Hosseini (2019) used the FIFA 11+ training program in a quasi-experimental study with male volleyball players and found significant improvements in agility, horizontal jump, and vertical leap. This highlights the efficacy of systematic training programs, such as FIFA 11+, in enhancing certain athletic skills [31]. Hale (2019) used a prospective experimental approach to show significant improvements in vertical jump metrics and agility performance in female youth volleyball athletes after an eight-week mixed strength and plyometric/agility conditioning program, demonstrating the efficacy of a holistic training program in improving leaping abilities and agility [32].

Roopchand-Martin et al. (2018) investigated innovative training modalities, such as dance video game training, and found significant gains in agility. This study demonstrates that unusual and engaging strategies can improve volleyball players' agility [33]. Ho et al. (2016) discovered that plyometric workouts improved agility, countermovement jump, and blocking agility, highlighting the usefulness of plyometric training in enhancing agility and vertical leap [34]. Jastrzbeski et al. (2014) found substantial increases in vertical jump heights and power generation with both high-intensity and low-intensity plyometric training, indicating that both modalities could improve leaping ability [35].

Gortsila (2013) assessed a 10-week volleyball training program on various surfaces and found substantial increases in the agility T-test and 505-test performance. This study demonstrates that training programs can improve agility, with the training surface influencing the results [36]. Stojanović and Jovanović (2012) observed that plyometric training significantly improved leaping agility and explosive leg strength [37].

Overall, the literature shows that a variety of training interventions, including plyometric exercises, core training, and novel approaches such as dance video games, can greatly improve volleyball players' agility, strength, and vertical jump abilities. These findings offer significant recommendations to coaches and athletes looking to improve their training routines and on-court performance. An ongoing study into the most effective training regimens adapted to certain volleyball player profiles is likely to develop and advance training procedures in the future.

Limitations

The systematic evaluation of the included papers on volleyball training treatments indicates numerous limitations. Many studies have small sample sizes and short intervention duration, limiting the validity and long-term consequences of the findings. The lack of diverse study populations and control groups, along with variations in training programs, makes it difficult to compare the effectiveness of interventions directly. Potential publication bias and the use of diverse outcome measures further complicate the synthesis of results. Additionally, the absence of blinding in some studies may introduce bias. Acknowledging these limitations highlights the need for more comprehensive, standardized, and rigorous research to understand better training intervention effects on volleyball players' athletic performance.

## Conclusions

The systematic review of these studies explores the impact of various training methods on volleyball players' agility, emphasizing their significance. While the interventions varied from traditional resistance training to innovative approaches, many studies reported positive effects on agility performance. Despite limitations like sample size constraints and variability in study populations, these findings underscore the need for tailored training programs to enhance volleyball players' agility.
